# Motivational network intervention to reduce substance use and increase supportive connections among formerly homeless emerging adults transitioning to housing: study protocol for a pilot randomized controlled trial

**DOI:** 10.1186/s13722-021-00227-9

**Published:** 2021-03-16

**Authors:** Joan S. Tucker, David P. Kennedy, Karen Chan Osilla, Daniela Golinelli

**Affiliations:** RAND Corporation, 1776 Main Street, Santa Monica, CA 90407 USA

**Keywords:** Substance use, Supportive connections, Intervention, Motivational interviewing, Social networks, Homeless, Young adults

## Abstract

**Background:**

Studies indicate high rates of substance use among youth experiencing homelessness (YEH). Further, the social networks of YEH, although multi-dimensional in composition, are largely comprised of other YEH, substance users, and individuals who do not provide the youth with tangible or emotional support. For YEH who have the opportunity to enter a housing program, helping them to reduce their substance use and strengthen their prosocial supportive connections during this critical transition period may increase their stability and reduce their risk of re-entering homelessness. The goal of this study is to pilot test a brief motivational network intervention (MNI), delivered by case managers, to help former YEH who have recently transitioned to a housing program reduce their substance use and strengthen their prosocial supportive connections.

**Methods/design:**

Up to 60 residents of housing programs in the Los Angeles area will be randomized to receive four sessions of usual case manager support or four sessions of case manager support + MNI. Each MNI session consists of three parts: (1) identifying two goals that are most important for the resident over the next year (e.g., get or keep a job, finish or stay in school, reduce substance use); (2) a network interview with the resident to capture network data pertaining to their interactions in the past 2 weeks; and (3) a discussion between the case manager and the resident of the resulting network visualizations, conducted in a Motivational Interviewing (MI) style, and what role the resident’s network may play in reaching their most important goals over the next year.

**Discussion:**

This study addresses a critical gap by pilot testing a computer-assisted MNI, delivered using MI techniques, that can help case managers work with recent YEH to reduce substance use and increase permanent supportive connections during the critical transitional period from homelessness to housing.

*Trial registration* ClinicalTrials.gov Identifier: NCT04637815. Registered November 10, 2020.

## Background

The goal of this study is to conduct a pilot evaluation of a motivational network intervention (MNI) to reduce substance use and strengthen supportive connections for recently homeless 18–25 year-olds who have transitioned to a housing program. The most recent point-in-time U.S. homeless count indicates that, on any given night, over 35,000 youth under age 25 are unaccompanied (without a parent or guardian present) and experiencing homelessness [1]. High rates of alcohol and drug use are consistently found in studies of these youth experiencing homelessness (YEH) [2–5], with an estimated 60–70% meeting lifetime criteria for a substance use disorder [6–9]. YEH tend to engage in substance use with social network members who themselves are involved in multiple risk behaviors [10]. In fact, the social environments of YEH, although multi-dimensional in composition, are largely comprised of other YEH, substance users, and individuals who are unemployed and disengaged from school [11]. Further, most of their relationships do not provide them with tangible or emotional support [11, 12]. For YEH who have the opportunity to enter a housing program, helping them to reduce their substance use and strengthen their prosocial supportive connections during this critical transition period may increase their stability and reduce their risk of re-entering homelessness.

### Social network influences on individuals experiencing homelessness

Social networks are naturally occurring groups of people that influence health and behaviors through a variety of mechanisms, such as provision of support, access to resources and information, social comparison, social rewards and sanctions, stress reduction, and socialization [13, 14]. Social networks can play positive and negative roles in the lives of people experiencing homelessness in terms of influencing the types and amount of social support they receive, their substance use and mental health, and even their transition out of homelessness [15–21]. After entering a housing program, it is likely that the social networks of recently homeless young people undergo a period of heightened volatility. Their improved housing status may put them into frequent contact with new social influences, and may also provide them with opportunities to reconnect with those who can provide them with ongoing support. At the same time, these young people may have developed strong connections with street-based peers and have reservations about severing ties, even with those who they realize may hamper their efforts at positive behavior change and stability. The transition out of homelessness and into a housing program provides a unique and critical window for a social network intervention to help these young people make positive changes in their social networks, thereby reducing their substance use and strengthening their prosocial supportive connections.

### Social network interventions using a personal network visualization may help increase awareness of social network systems and provide insight into possibilities for change

The personal network approach defines the composition and structure of a social network of one individual [22–25]. Personal network approaches have been used to study substance use and other risk behaviors among individuals experiencing homelessness, including YEH [5, 17, 26]. Personal network data differ from more traditional social network data (“complete” or “sociocentric”) because personal network data are collected from individuals about their perceptions of their own social environments rather than their connections to members of a particular group. The advantage of a personal network approach is that most interventions focus on delivering treatment to individuals rather than groups; therefore, enhancing existing interventions with a focus on personal networks is a way to include a focus on social networks without requiring a design that targets a bounded group. Understanding personal networks is important because the social network of one individual operates as a complex system with emergent properties that affect the focal individual [27]. Personal network interviews enable the visualization of the immediate social network of the focal individual [28–30]. Personal network visualizations allow for display of both compositional and structural features. Compositional features include the quantity of types of people in the network, such as the number of family members, supportive network members, or drug use partners. Structural features include the quantity and configurations of connections among people in the network, such as the density of the network overall (ratio of existing connections to the maximum possible number of connections) or the centrality of particular network members within a network (e.g. the number of connections between one individual and other members of the network). Social network visualizations are not new to health-related interventions, as visualization tools have been used by social workers to provide feedback to families about health and social support [31, 32]. However, the most commonly used techniques only provide information about network composition and do not incorporate structure.

Our team has pilot tested a network visualization intervention that incorporates structure to help older adults who recently transitioned from homelessness to a housing program reduce their substance use [33, 34]. Results demonstrated beneficial effects on readiness to change, abstinence self-efficacy, and substance use [35]. However, the intervention was not delivered by case managers directly to residents, and YEH may have different social challenges that should be explicitly addressed. Case managers who work with young residents also have a need to understand the personal networks of the youth they support to have discussions with them about their social challenges and opportunities for support from their networks. Discussions between case managers and residents about their networks with a focus on network visualizations that highlight positive and negative aspects of their social environments may greatly improve these discussions. The visualizations enable case managers and residents to discuss subtle aspects of their network composition and structure that may be difficult to articulate without a visual aid.

### Conceptual framework

**Fig. 1:**
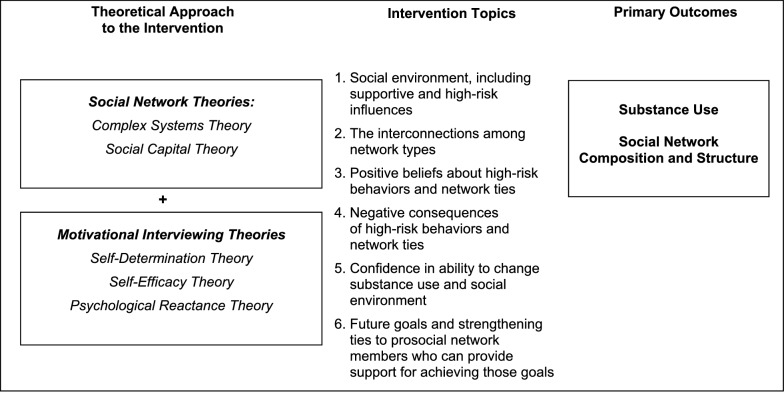
Conceptual framework

MNIs are grounded in theories underlying social network (Complex Systems and Social Capital Theories) and Motivational Interviewing (Self-Determination, Psychological Reactance, and Self-Efficacy Theories) approaches (see Fig. 1). The MNI we are evaluating in this study targets social network composition and structure and the relationship of these to the resident’s substance use and the support that is available from others to help them reach their goals. Complex Systems Theory as applied to social networks assumes that a set of social relationships between individuals in a group has emergent properties that would not be apparent in an examination of the individual parts of a larger social system [36, 37]. The theory also suggests that changes made in one area of the system may have effects that flow throughout the rest of the system and that approaches to change should consider the potential impact on the whole system. Social Capital Theory identifies an emergent property of the social resources in a network, such as information channels, norms and expectations/obligations that impact people’s lives [38–43]. Social capital can have positive or negative impacts on health and network resources are either amplified or dampened depending on how and with whom links are formed [44, 45]. MI is an evidence-based intervention style [46, 47] that has been used to reduce substance abuse among young people [48], including YEH [49]. MI moves people towards change through strategies such as expressing empathy (e.g., acceptance facilitates change), developing discrepancy (e.g., letting the client present arguments for change), rolling with resistance (e.g., avoiding arguing), and supporting self-efficacy (i.e., a person’s belief in the possibility of change). MI has its foundation in Self-Determination Theory which emphasizes client autonomy and innate capacity for growth and change [50], Self-Efficacy Theory which indicates that people with more confidence in their ability to change their behavior are more likely to change [51], and Psychological Reactance Theory which posits that when people feel undue pressure to give up a particular action, they feel that their freedom is threatened [52].

Together, these theories suggest that an intervention that presents YEH who have recently transitioned to a housing program with personalized network information using a MI style may empower them to transition away from high-risk social networks and toward low-risk, supportive social networks. For example, in this study, we will train case managers to introduce the MNI by emphasizing autonomy with statements such as “The goal of this interview is to give you information so that you can better understand and make decisions about your social life. What you do with this information is totally up to you.” To build self-efficacy, case managers are trained to identify statements of change and to encourage change talk by asking residents to explain their reasons for wanting change and to reinforce this through encouragement. Case managers also ask residents to talk about practical steps they can take to initiate changes in their social networks. This strategy is consistent with MI by eliciting and strengthening confidence talk or the language associated with self-confidence to change. Having the resident verbalize his/her reasons for change and the steps to initiate change may reduce perceived barriers to change and increase self-efficacy. In addition, this type of discussion can bring to light challenges in making a change, which the case manager can then help to problem solve with the resident.

### The present study

The goal of this pilot study is to evaluate, through a small randomized controlled trial (Stage 1b), the added benefit of incorporating the MNI into case management for 18–25 year old residents of housing programs. We will pilot test the MNI with up to *N* = 60 residents. We hypothesize that residents receiving the MNI as part of case management (*n* = 30) will show more positive changes in their substance use behaviors and the composition and structure of their personal networks (i.e., greater and more central network members who are low-risk and supportive influences) over a 3-month follow-up period compared to residents receiving usual case management (*n* = 30).

## Methods/design

### Participants

Individuals will be recruited through a nonprofit organization in Los Angeles County that provides a comprehensive continuum of care to YEH that includes free emergency resources such as food and clothing in combination with case management, health, vocational, educational, therapeutic, and housing services. Individuals will be eligible for the study if they: (1) are age 18 to 25; (2) are in a housing program serving YEH; (3) screen positive for past-year harmful substance use using the Global Appraisal of Individual Needs—Short Screener (GAIN-SS; [53] score of 3 or higher); (4) have used alcohol or drugs in the past 30 days; and (5) are willing to provide contact information for themselves and others who will know how to reach them so the resident can be contacted to complete a follow-up survey 3 months later. Individuals will be ineligible if they: (1) are not able to speak and understand English; and (2) have been in the housing program for longer than one year. We plan to enroll up to 60 participants at baseline, who are expected to represent the demographics of the broader population of 18–25 year-olds experiencing homelessness in Los Angeles County. All participants, regardless of condition, will be eligible for all services provided by the housing program. Study materials and procedures have been approved by RAND’s Human Subjects Protection Committee and a Certificate of Confidentiality from the National Institutes of Health will protect data from subpoena. An independent Data and Safety Monitoring Board with relevant expertise will meet regularly during the field period to monitor study procedures, progress, and any adverse events.

### Procedures

Case managers will individually contact residents of their housing programs and screen for substance use. Case managers will ask those who screen positive if they are willing to be contacted by RAND about the study and, for those who agree, will collect contact information from the resident to be shared with the research team via a secure file sharing service. Residents who consent to be contacted will be assigned a unique study ID number, complete an eligibility screener for the study, and (if eligible and interested in participating) asked to provide informed consent and detailed tracking/locating information, and complete the baseline survey via computer-assisted telephone interview. Survey responses will be entered by the research team member directly into the EgoWeb 2.0 application installed on a virtual webserver. Access to the application is protected by password and all data are secured with strong encryption. The only identifying information entered along with baseline responses will be the respondent ID number and no other personal information (names, phone numbers, etc.) will be recorded during baseline.

**Fig. 2:**
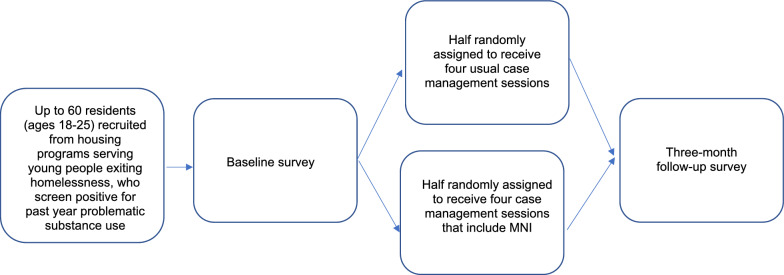
Randomized controlled trial study flow

After completing the baseline survey, residents will be randomly assigned to usual case manager support or case manager support + MNI using a stratified [by housing type (group residential facility vs. other sites) and time since entering housing program (past 2 weeks vs. longer)] permuted block randomization strategy. RAND will be responsible for the randomization and will inform the case manager that recruited the participant of the randomization results so that s/he knows whether to conduct the MNI sessions. The randomization sequence will be generated by our project statistician and kept hidden from other team members so that the assignment to the two conditions cannot be gamed. A follow-up survey will be administered 3 months after enrollment via computer-assisted telephone interview, with the outcome assessor blinded to the participant’s study condition. Plans to promote participant retention in the study include making an interim contact with each resident 6 weeks after baseline to obtain updated contact information and a graduated incentive system for completing the surveys. Figure 2 depicts participant flow through the study.

### Description of the intervention

The MNI is designed to be delivered by a trained case manager as part of their regular case management meetings with residents. Case managers are provided with a handbook that provides step-by-step instructions for using the MNI and watch a 30-min video demonstration of an MNI session. They also receive a half-day in-person training on Motivational Interviewing (MI) techniques and a 2-h training on using the EgoWeb 2.0 platform (see egoweb.info) to deliver the MNI. Prior to using the tool with residents, case managers participate in supervised role-plays conducting the intervention a minimum of three times and receive additional training until the case manager demonstrates proficiency in conducting an MNI session.

The MNI consists of four sessions that build on each other and can be delivered face-to-face or virtually. Similar to the baseline and follow-up assessments, MNI session responses will be entered into the same secure EgoWeb 2.0 application along with the same participant ID. Each MNI session lasts approximately 30 min and consists of three parts: (1) identifying two goals that are most important for the resident over the next year (e.g., get or keep a job, finish or stay in school, have own place to live, reduce substance use, strengthen relationships); (2) a network interview with the resident to capture network data pertaining to their interactions in the past 2 weeks; and (3) a discussion between the case manager and the resident of the resulting network visualizations, conducted in a MI style, and what role the resident’s network may play in reaching their most important goals over the next year. We discuss the latter two parts of the session in more detail below.

*Network interview* Case managers read the network questions from EgoWeb 2.0 verbatim. Answers to these questions provide raw data to generate network visualizations. These structured network interview questions are an abbreviated set of the questions asked at baseline (described below), cover the timeframe between the baseline and first session (or between visits in subsequent sessions), and consist of three components. The interview is best conducted when the participant can also see the EgoWeb 2.0 screen to facilitate easier responding. The first component is a network name generator (e.g., “Please name 15 people, who are at least 18 years old, who you have talked to the most over the past 2 weeks, either in person or over the phone, or by texting, emailing, etc.”). The second component is network composition questions focusing on support receipt (e.g., Consider who could help you reach your goals in the next year. Pick the option that best describes each person: (a) I could go to them if I needed support reaching my goals in the next year; (b) I could go to them now for support, but not sure I can count on them in the future; (c) I would not go to them for support in reaching my goals—either now or in the future) and substance use (e.g., Pick the option that best describes each person’s alcohol and drug use; if you’re not sure, give your best guess: (a) They will probably not use in the next 2 weeks; (b) They will probably use in the next 2 weeks, but not with me; (c) They will probably use in the next 2 weeks, and I would probably use my regular amount or less with them; and (d) they will probably use in the next 2 weeks, and I would probably use more than usual with them). The third component is network structure items (e.g., Let’s talk about who in your network knows each other. For each pair of people, pick the best option: (a) they don’t know each other; (b) they know each other but did not connect recently; and (c) they know each other and connected recently).

*Network discussion* Once all network questions have been asked and answered, case managers will transition to discussing the network visualizations generated by EgoWeb 2.0. They will show the resident a series of three network diagrams customized for the resident based on the responses they provided in the network interview and use a MI style to discuss the resident’s reactions. Each diagram will focus on a particular aspect of the network and will highlight key network members (“alters” in social network terminology) and subgroups by varying colors, node sizes, thickness of lines, etc. The initial diagram will present the network structure (drawing visual attention to subgroups, highly central members, isolated members, etc.) and subsequent diagrams will highlight social support (consistent sources of support for achieving their goals) and substance use (who is likely to use substances, who is a negative influence on the resident’s substance use) in the network. Case managers will explore the pros and cons of residents’ current social network composition and structure, discuss their willingness to make changes to their substance use and what steps they can make in the next two weeks, and changes they want to make in their social networks in the next two weeks (e.g., spending more or less time with certain network members; making new connections). Case managers can use the MNI to record notes on the statements the resident makes regarding their goals and the steps they are willing to take towards modifying their substance use and social network relationships over the next two weeks.

The three subsequent sessions cover the time periods between sessions and repeat the network interview and discussion. The MNI displays the names of network members mentioned in previous sessions, but participants are able to name new network members. These sessions begin with a discussion of the residents’ stated goals from the previous session. Discussions of the visualizations focus on the ways in which the network changed between sessions, including discussing alters they either started or stopped interacting with since the previous MNI session and discussing what has not changed since the last interview. Case managers review notes from previous interviews and ask follow-up questions about attempts at change and encourage steps that residents are taking toward meeting their goals.

The in-depth discussions of the social environment of the resident and the documentation of the network composition/structure and stated goals about change are designed to enhance the supportive relationship between the case manager and resident beyond the individual MNI sessions. The network visualizations resulting from the network interviews provide both case managers and residents with a tangible output on which to focus discussions and make abstract discussions of social life, relationships, and social change more concrete. They also provide documentation of the personal goals and aspirations of the residents across sessions and facilitate discussions of successful attempts at change to allow for reinforcement during MNI sessions and other interactions.

### Description of usual care condition

Since we are interested in evaluating the added benefit of the MNI over usual case management, residents in the control condition will be asked to meet with their case manager four times (once every two weeks) during their participation in the study. These meetings are anticipated to last approximately 30–60 min and will be customized to the residents’ needs.

### Analytic plan

The major goal of the study is to assess intervention feasibility, acceptability and promise; estimate the variability of measures in the study population; and obtain preliminary estimates of intervention effect sizes. The analyses will be primarily descriptive given the small sample size; sophisticated modeling and adjustments for non-response might not be possible. We will estimate the outcomes’ variability in this population and more generally assess the hypothesized trends to determine the intervention’s promise. Analyses will use the standard intent-to-treat (ITT) approach such that residents will be analyzed as belonging to the group they were randomized to, regardless of their compliance, because excluding those who do not complete the MNI would bias results in favor of MNI, increasing type I errors.

We will first examine variable distributions from both time points to assess missing data patterns, and will use a model-based multiple imputation approach for these missing data where appropriate. Although participants will be randomly assigned to condition, imbalance between the two groups on some baseline characteristics may occur. Such differences can contribute to the variability in estimates of intervention effects, weakening the power of the tests. In all relevant analyses we will control for those variables that exhibit baseline imbalance between the two groups by including them in the models. This pilot study may not have sufficient power to demonstrate statistically significant effects or fit sophisticated models. However, we will be able to obtain intervention effect estimates using a difference in differences (DID) approach. The DID approach is well-suited to the data generated by the adopted study design: for every participant we will have a pre- and post-intervention observation and a randomized group indicator (intervention or control). We will implement the DID approach by fitting a mixed-effects model in which participants are treated as random effects while time, the intervention group indicator, and their interaction are treated as fixed effects. This modeling approach accounts properly for the two repeated measures’ correlation on each participant and tends to produce efficient intervention effect estimates.

### Main outcomes of the intervention

The main outcome variables will be past month substance use and network composition at the 3-month follow-up. Using items based on the Monitoring the Future survey [54], we will assess the number of days, in the past 30 days, participants engaged in the use of various substances, including alcohol use, heavy alcohol use (defined as 5 or more drinks in a row for men, 4 or more drinks in a row for women), cannabis use, non-medical prescription drug use, and use of several types of illicit drugs (e.g., heroin, methamphetamine, hallucinogens). We will also ask about the number of drinks typically consumed on drinking days in the past month. Based on this information we will be able to calculate number of days of drug use, number of days of alcohol use, number of days of heavy alcohol use, and number of drinks consumed on drinking days.

Network composition will be assessed by asking participants to “think about people you have connected with the most over the past 4 weeks, either in person or over the phone, or by texting, emailing… things like that.” The participant will name 15 people (by first name, initials, a nickname, or other description) who are at least 18 years old and then answer a series of questions about each person.These questions will include: whether this person is a family member; how often they connected with this person in the past 4 weeks; how often they used alcohol or drugs with this person in the past 4 weeks; if applicable, whether they tended to engage in more, less, or about the same substance use as usual when they were with this person; and whether they would go to this person for support in meeting the goals they have set for themselves, for emotional support or encouragement, and for money transportation, food, or other items that they need (separate items). Network composition measures will include the proportion of supportive ties, the proportion of risky ties in terms of substance use, and how central various types of network members are in the network using standard social network centrality measures [23, 55].

### Limitations and alternative methods considered

The main study limitations include: (1) A small sample size limits our ability to detect statistically meaningful differences in our outcomes; (2) Greater participant understanding of their networks may influence how they answer questions about their networks in the post MNI assessment, limiting our ability to separate changes in networks from changes in perceptions of networks; and (3) The completion of the baseline network interview may also have unexpected effects on the control group even if they have not seen a visualization of their network. Insights from this feasibility study will inform how to overcome these limitations in a large sample Stage 2 effectiveness study. Note that all case managers will be trained to deliver both the MNI and usual care case management conditions, rather than randomly assigning case managers to one condition or the other. While there may be spillover across conditions in the use of MI to deliver case management, it is not expected that the network interview and resulting discussion of network visualizations (i.e., the “active ingredients” of the MNI case management) can be replicated in the usual care condition since it does not involve use of the EgoWeb 2.0 software. We will be audiorecording all case management sessions (in both the MNI and usual case management conditions) and listening to them on a weekly basis to monitor fidelity to treatment assignment. We will meet regularly with the case managers to provide feedback and (if necessary) retraining on study protocols.

## Conclusions

In summary, YEH are a high risk and vulnerable population who have unique needs, especially as they transition to housing. As discussed above, studies by our team and others have shown how the social networks of individuals experiencing homelessness are multi-faceted and influence a range of health behaviors (e.g., substance use) and related outcomes (e.g., successful transition out of homelessness). A social network intervention for YEH transitioning to housing programs may be critically important to reduce their substance use and increase their prosocial supportive connections—both of which may increase the likelihood of long-term housing stability. Using MI is an ideal fit for a social network intervention with young people as it has been successfully used to reduce substance use in this and other populations. If successful, this study may provide a template for the development of social network-based health interventions that can target a diverse array of outcomes and benefit a wide variety of populations.

## Data Availability

Once collected, deidentified data from this study will be available from the corresponding author on reasonable request 1 year after all aims of the project are completed. Requestors of data will be asked to complete a data-sharing agreement that provides for (1) a commitment to using the data only for research purposes and not to identify any individual participant; (2) a commitment to securing the data using appropriate computer technology; and (3) a commitment to destroying or returning the data after analyses are completed.

## Abbreviations

MNI: Motivational network intervention.
MI: Motivational interviewing.
YEH: Young people experiencing homelessness.
